# Mouse models for dominant dystrophic epidermolysis bullosa carrying common human point mutations recapitulate the human disease

**DOI:** 10.1242/dmm.048082

**Published:** 2021-06-04

**Authors:** Blake R. C. Smith, Alexander Nyström, Cameron J. Nowell, Ingrid Hausser, Christine Gretzmeier, Susan J. Robertson, George A. Varigos, Cristina Has, Johannes S. Kern, Ken C. Pang

**Affiliations:** 1 Murdoch Children's Research Institute, Parkville, VIC 3052, Australia; 2 Department of Paediatrics, University of Melbourne, Parkville, VIC 3052, Australia; 3 The Walter and Eliza Hall Institute of Medical Research, Parkville, VIC 3052, Australia; 4 Department of Dermatology, Medical Center - University of Freiburg, Faculty of Medicine, University of Freiburg, Freiburg 79104, Germany; 5 Monash Institute of Pharmaceutical Sciences, Monash University, Parkville, VIC 3052, Australia; 6 Institute of Pathology, University Hospital Heidelberg, Heidelberg 69120, Germany; 7 Dermatology Department, Faculty of Medicine, Dentistry and Health Sciences, The Royal Melbourne Hospital, University of Melbourne, Parkville, VIC 3050, Australia; 8 Royal Children's Hospital, Parkville, VIC 3052, Australia

**Keywords:** Epidermolysis bullosa, Mouse model, Skin, Blistering

## Abstract

Heterozygous missense mutations in the human *COL7A1* gene – coding for collagen VII – lead to the rare, dominantly inherited skin disorder dominant dystrophic epidermolysis bullosa (DDEB), which is characterised by skin fragility, blistering, scarring and nail dystrophy. To better understand the pathophysiology of DDEB and develop more effective treatments, suitable mouse models for DDEB are required but to date none have existed. We identified the two most common *COL7A1* mutations in DDEB patients (p.G2034R and p.G2043R) and used CRISPR-Cas9 to introduce the corresponding mutations into mouse *Col7a1* (p.G2028R and p.G2037R). Dominant inheritance of either of these two alleles results in a phenotype that closely resembles that seen in DDEB patients. Specifically, mice carrying these alleles show recurrent blistering that is first observed transiently around the mouth and paws in the early neonatal period and then again around the digits from 5-10 weeks of age. Histologically, the mice show micro-blistering and reduced collagen VII immunostaining. Biochemically, collagen VII from these mice displays reduced thermal stability, which we also observed to be the case for DDEB patients carrying the analogous mutations. Unlike previous rodent models of epidermolysis bullosa, which frequently show early lethality and severe disease, these mouse models, which to our knowledge are the first for DDEB, show no reduction in growth and survival, and – together with a relatively mild phenotype – represent a practically and ethically tractable tool for better understanding and treating epidermolysis bullosa.

This article has an associated First Person interview with the first author of the paper.

## Abstract (precis)

**Summary:** We developed mouse models for the blistering genetic skin disorder dominant dystrophic epidermolysis bullosa (DDEB) by introducing mutations into mouse *Col7a1*. These models should help to improve the understanding and treatment of DDEB.

## INTRODUCTION

Epidermolysis bullosa (EB) is a group of genetic skin fragility conditions caused by disruption of epithelial adhesion proteins in the skin. Mutations in the gene coding for collagen VII (*COL7A1*) lead to a disease type known as dystrophic epidermolysis bullosa (DEB), which can be inherited in either a dominant (DDEB) or recessive (RDEB) manner (Fine et al., 2014; Has et al., 2020). Collagen VII forms anchoring fibrils, critical structural components of the skin's basement membrane that anchor it to the dermis (Sakai et al., 1986). Collagen VII contains a triple-helical domain, which consists largely of a G-X-Y repeating motif (where G is glycine, and X and Y are different amino acids) that is critical for its structural integrity. Autosomal-dominant mutations causing DDEB are manifold, but typically involve glycine substitutions that disrupt this G-X-Y motif and lead to dominant-negative interference during anchoring fibril assembly and reduced triple-helix stability. Owing to this reduced stability, the structural integrity of collagen VII and the basement membrane are compromised (Chung and Uitto, 2010). The epidermis and dermis thus separate more readily. This separation can occur spontaneously or in response to triggers such as mechanical friction, humidity or temperature change. Clinically, this manifests as skin and mucosal blisters and erosions, which heal with atrophic scarring, milia formation, nail dystrophy and alopecia. Over time, repeated epithelial wounding and scarring can lead to joint contractures and mucosal strictures (Bruckner-Tuderman and Has, 2014; Watkins, 2016).

To better understand the pathophysiology of epidermolysis bullosa (EB) and develop more effective treatments, a number of pre-clinical animal models of different EB subtypes have been developed. These include knockouts or conditional knockouts (Nyström et al., 2013a; Heinonen et al., 1999) and hypomorphic mouse models of RDEB (Fritsch et al., 2008), for which clinical presentation is generally more severe than that for DDEB, which arise due to mutations causing complete or almost complete loss of collagen VII. Although a spontaneously arising mutation in *Col7a1* led to the existence of a rat model for DDEB (Nyström et al., 2013b), there is currently no published mouse model of DDEB (Bruckner-Tuderman et al., 2010; Has and Nyström, 2015). This is problematic, because the development of novel therapies for DDEB requires different strategies from that for RDEB. Specifically, although methods such as gene therapy, protein replacement or stem cell transplantation offer hope of providing a source of functional collagen VII for RDEB, they will not remove the defective collagen VII of DDEB that interferes with the assembly of normal anchoring fibrils.

The aim of this study was to develop the first mouse model of DDEB. Traditionally, generating a new mouse model presents many challenges, requiring significant investment of time and effort. However, creating genetically modified mice has become easier with the advent of CRISPR-Cas9-mediated genome editing, which allows for targeted mutations – both deletions and point mutations – to be readily introduced into specific genes of interest.

Here, we describe the successful development and characterisation of multiple novel mouse models of DDEB via CRISPR-Cas9. Using a publicly available database, we selected the two most commonly reported *COL7A1* mutations resulting in DDEB – both of which occur in a region that is highly conserved between human and mouse *COL7A1* genes – and introduced these separately into mouse embryos. These mouse models closely recapitulate the dominantly inherited blistering phenotype of patients with DDEB and represent new pre-clinical models for improving our understanding and treatment of DDEB.

## RESULTS

### Selection of targeted DDEB mutations

**Fig. 1.**
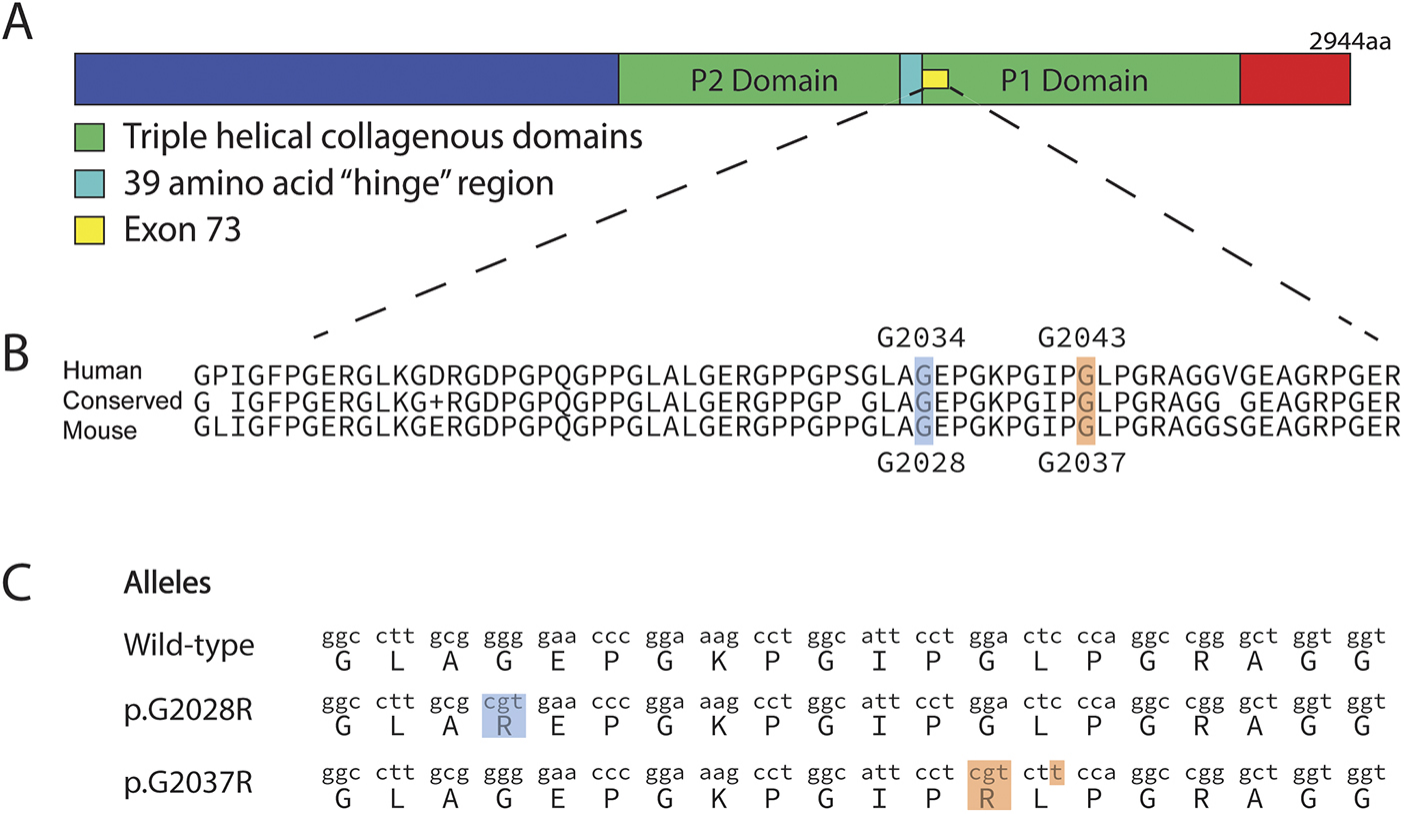
**Targeted *Col7a1* mutations introduced via CRISPR-Cas9 editing.** (A) Collagen VII has a triple-helical domain, interrupted by a 39-amino acid ‘hinge’ region. Exon 73 codes for the region directly upstream of the hinge region. aa, amino acids. (B) Most mutations causing dominant dystrophic epidermolysis bullosa (DDEB) are within exon 73, which is highly conserved between human and mouse. The two most frequently mutated amino acids in human DDEB are G2034 and G2043. These glycines (and the amino acids surrounding them) are highly conserved and correspond to G2028 and G2037, respectively, in mice. (C) Using CRISPR-Cas9, we introduced *Col7a1* mutations (p.G2037R and p.G2028R) in mice (designated *Col7a1*^em1Kepa^ and *Col7a1*^em2Kepa^, respectively).

The publicly available DEB Register collects DEB patient data globally, including information on clinical features and specific mutations (www.deb-central.org/). Using this registry, we identified that the majority of mutations reported in DDEB occur within exon 73, just after the hinge region of the triple-helical domain and within the G-X-Y repeating motif. In particular, two mutations that cause replacement of the glycine residue at amino acid positions 2034 and 2043 account for the majority of DDEB mutations (Fig. 1A,B). Because this region is highly conserved between the mouse and human genomes, we decided to directly target the homologous glycine (G) residues in the mouse genome (at amino acid positions 2028 and 2037, respectively) and replace them with arginine (R) residues.

### Generation of novel *Col7a1* alleles using CRISPR-Cas9

Using CRISPR-Cas9-mediated genome editing, we introduced two separate mutations, p.G2028R and p.G2037R, into the genomes of early-stage mouse embryos and confirmed the success of this procedure by next-generation sequencing of affected pups. In addition to these two new alleles, which we designated *Col7a1*^em1Kepa^ and *Col7a1*^em2Kepa^ (Fig. 1C), three other novel *Col7a1* alleles were inadvertently generated as a by-product of the CRISPR-Cas9 endonuclease and comprised small, in-frame microdeletions of six, 11 and 15 amino acids (p.I2035_G2040del, p.A2027_G2037del and p.G2028_A2042del), which we designated *Col7a1*^em3Kepa^, *Col7a1*^em4Kepa^ and *Col7a1*^em5Kepa^, respectively (Fig. S1).

### Each novel *Col7a1* mouse line displays dominantly inherited blistering

Male mice hemizygous for each of these novel *Col7a1* alleles were bred to C57Bl/6J female mice, and genotyping of the resultant offspring at 3 weeks of age showed that mice carrying the affected alleles were recovered alive at the expected 1 in 2 Mendelian ratio (Table S1).

**Fig. 2.**
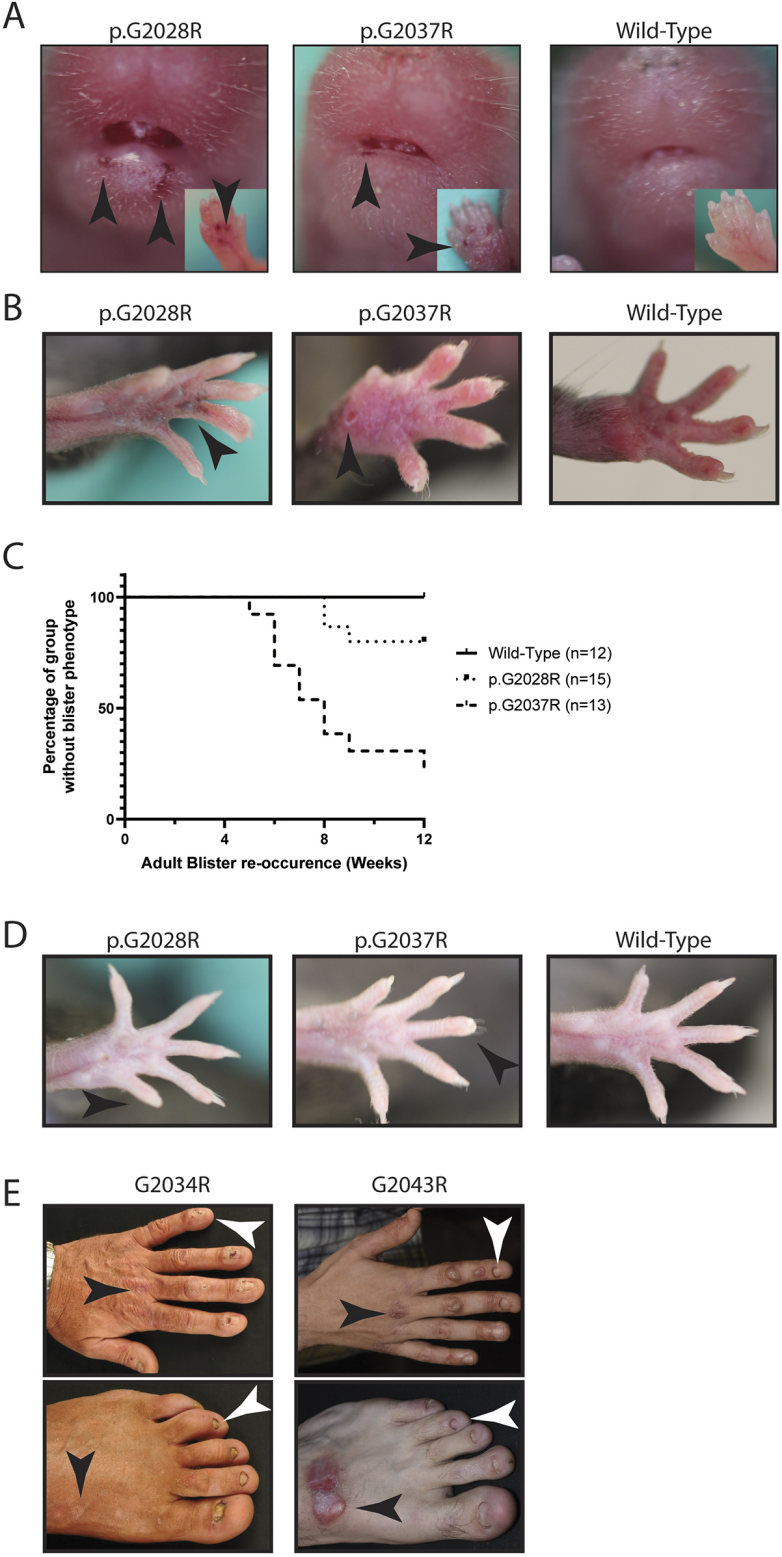
**The two targeted mutations lead to blistering with varying penetrance of re-occurrence.** (A) One-day-old mouse pups carrying the targeted alleles show varying amounts of blisters (indicated by black arrowheads) around the mouth and on the paws (insets). (B) These blisters (indicated by black arrowheads) fade by the time the mice are 1 week old and re-occur as the mice age, affecting the paws and generally becoming visible from 6-10 weeks of age. (C) Graph showing adult blister occurrence with different patterns of onset between p.G2028R and p.G2037R alleles, with the earliest occurrence at 5 weeks of age. Mice with the p.G2028R mutation show a later onset of blistering and lower penetrance, whereas mice with the p.G2037R mutation have earlier onset and higher penetrance, with most mice affected by 12 weeks of age. (D) At 12 weeks of age, the mice start to lose nails (indicated by black arrowheads). (E) Patients with the p.G2034R (mouse p.G2028R) and p.G2043R (mouse p.G2037R) mutations display nail dystrophy and loss (indicated by white arrowheads) (similar to that of the mice shown in D) as well as acral blistering and scarring (indicated by black arrowheads) (similar to that of the mice shown in A and B).

Visual inspection of these mice revealed mild to moderate blistering that was dominantly inherited. At postnatal day (P)1, mice carrying the targeted mutations (Fig. 2A) or the microdeletion alleles (Fig. S2A) displayed blisters on the paws and around the mouth, which disappeared by P7. Blisters then re-appeared around the digits from ∼5 to 10 weeks of age, depending on the underlying allele (Fig. 2B,C; Fig. S2B,C), with the p.G2037R point mutation showing much greater penetrance than p.G2028R among the two targeted mutations. By 12 weeks of age, loss of nails also started to be evident (Fig. 2D; Fig. S2D), with the frequency again allele dependent. Taken together, the macroscopic changes observed among these mice are directly reminiscent of those seen in patients with p.G2034R and p.G2043R (corresponding to mouse p.G2028R and p.G2037R, respectively) mutations, who similarly show signs of blistering and nail loss (Fig. 2E). Of note, mice carrying these novel *Col7a1* alleles showed normal weight gain during development, and there were no signs of increased mortality even up to 6 months of age, with 3.0% of adult mice (nine of 300) requiring euthanasia due to ill health (most commonly malocclusion), a rate consistent with the known survival rates of wild-type C57BL/6 animals (Turturro et al., 2002).

### *Col7a1* mutations lead to microscopic changes in the skin

**Fig. 3.**
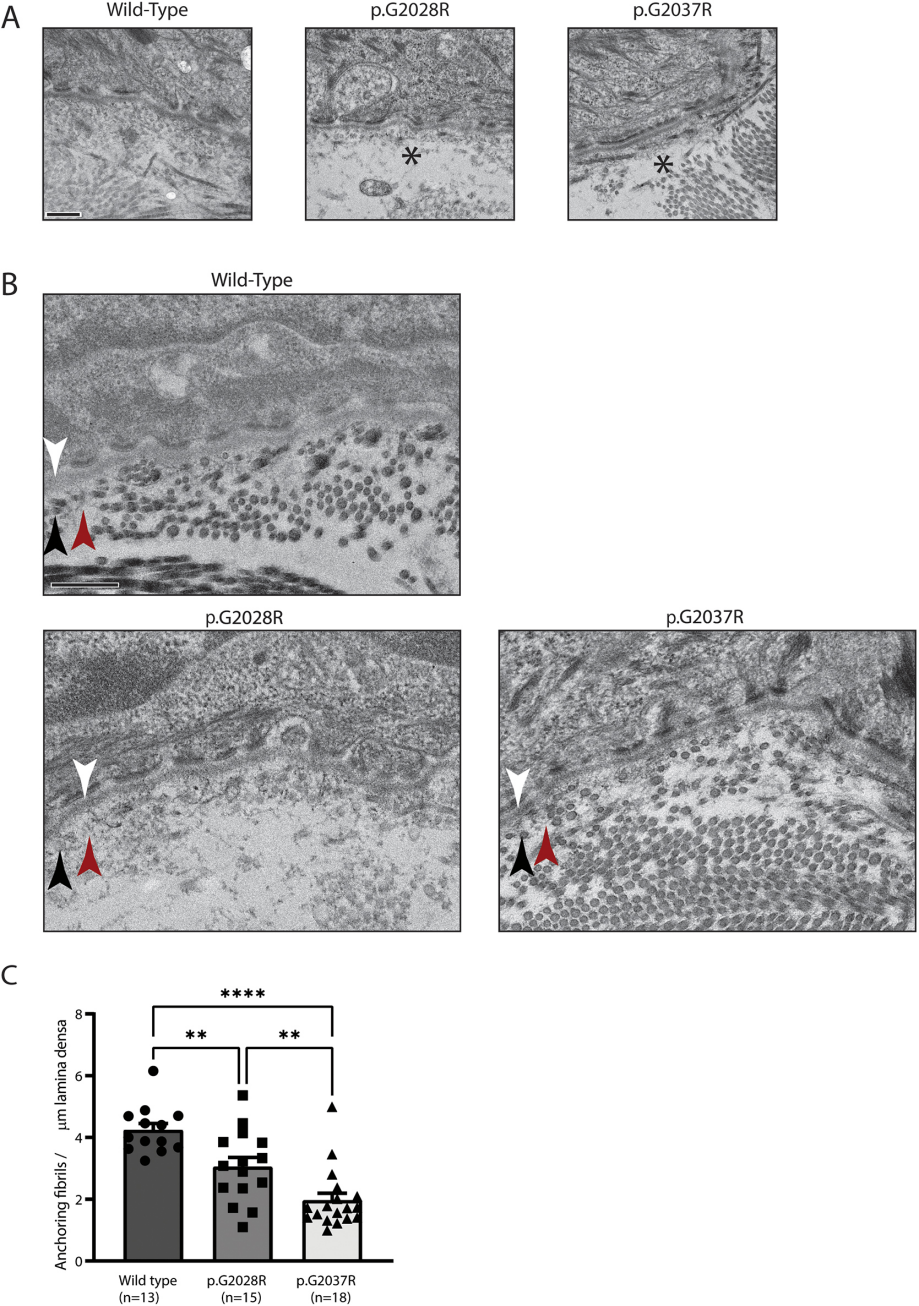
**Electron microscopy reveals putative separation and reduced anchoring fibrils.** (A) Electron microscopy of 12-week-old back skin shows putative separation below the lamina densa of the epidermal basement membrane, indicated by asterisks. Data are representative of multiple fields from a single biological replicate. Scale bar: 400 nm. (B) Electron microscopy also shows possible changes to the anchoring fibrils (indicated by red arrowheads) that sit below the lamina densa (indicated by white arrowheads) and among the interstitial collagen fibres (indicated by black arrowheads). Data are representative of multiple fields from a single biological replicate. Scale bar: 400 nm. (C) Quantification of anchoring fibrils per µm lamina densa, showing a reduction in both mutant mice. Number of random fields per genotype is indicated in the figure. Analysis by one-way ANOVA, **P<0.01, ****P<0.0001.

In addition to these macroscopic changes, light microscopy of Haematoxylin and Eosin (H&E)-stained skin sections taken from the back at 12 weeks of age revealed the presence of microscopic blisters at the dermal-epidermal junction of mice carrying the targeted mutations (Fig. 3A) as well as the microdeletion alleles (Fig. S3A).

**Fig. 4.**
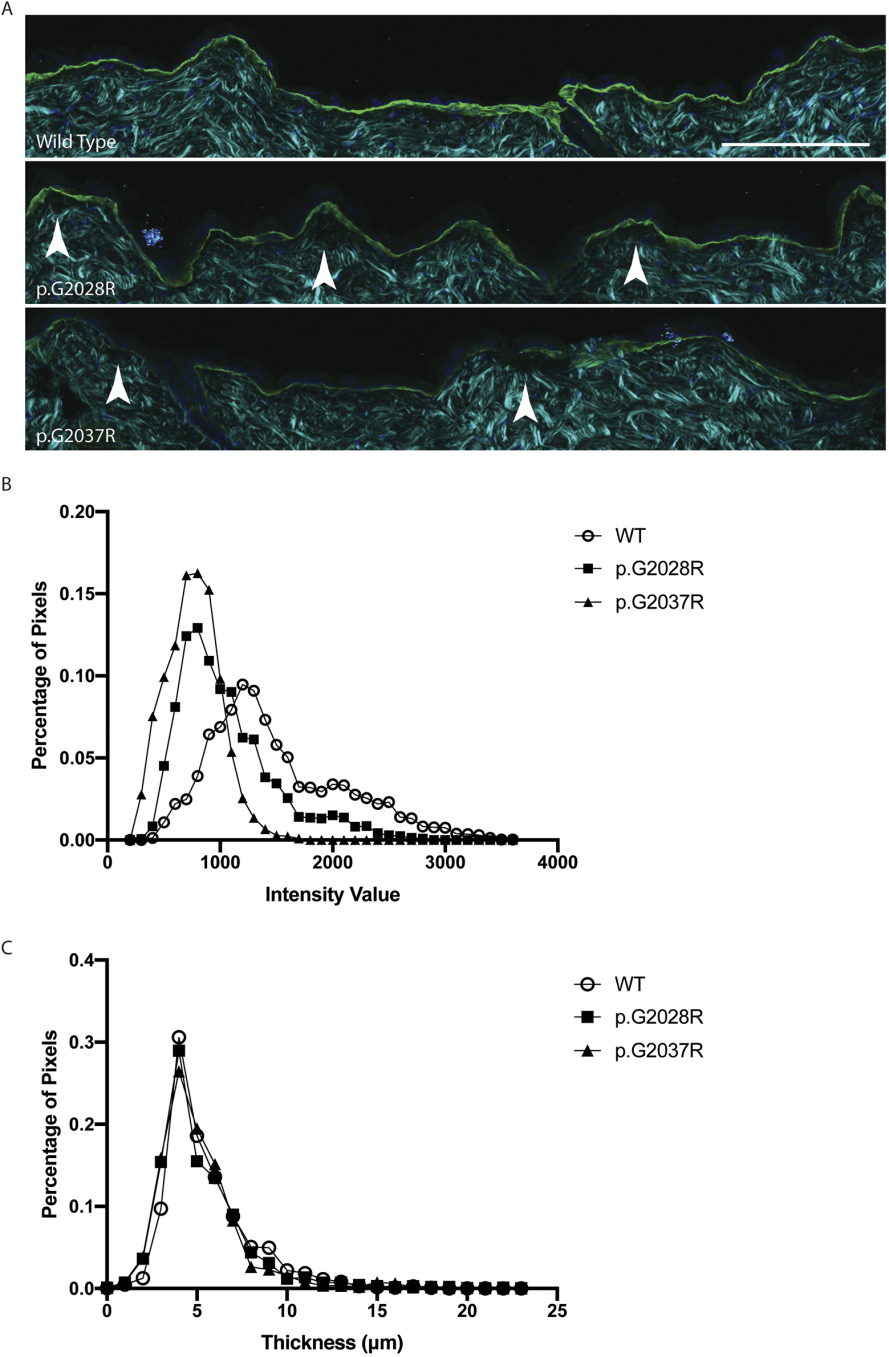
**Immunofluorescence staining shows changes in both collagen VII expression and basement membrane structure.** (A) Immunofluorescent staining of collagen VII (green) and DAPI (dark blue) in conjunction with second-harmonic imaging (cyan), which shows the structure of ordered collagens, in sections from 12-week-old mouse back skin. Changes in the intensity and thickness of collagen VII in the mutant sections are apparent when compared with wild-type skin, as is the presence of micro blisters (indicated by white arrowheads) below the basement membrane. Scale bar: 100 μm. (B) Analysis of the stained sections (five images per replicate, from three biological replicates per genotype) shows that the intensity of collagen VII staining varies across the mutants, with p.G2028R, and p.G2037R showing slightly less intensity than wild type (WT). (C) Analysis of the thickness of collagen VII staining shows that the p.G2028R and p.G2037R have a similar thickness to wild type.

To better understand the structural basis for the macroscopic changes observed above, electron microscopy (EM) and immunostaining for collagen VII was performed on skin sections from the backs of 12-week-old mice. EM sections taken from mice carrying the two targeted mutations showed putative separation below the epidermal basement membrane (Fig. 3A) and were suggestive of possible disruption of the anchoring fibrils (Fig. 3B). Formal quantification of the latter showed a significantly lower number of anchoring fibrils compared to wild-type tissues (Fig. 3C), with skin from the p.G2037R animals most affected. In contrast, we could not discern consistent ultrastructural differences between wild-type mice and those carrying the microdeletion alleles (Anton-Lamprecht, 1992; Chmel et al., 2018; Winberg et al., 1997) (Fig. S3). Meanwhile, immunostaining for collagen VII showed a reduction in staining intensity for each of the two targeted alleles compared to wild type (Fig. 4A). Formal quantification of collagen VII staining intensity (Fig. 4B) confirmed this impression, and revealed that skin carrying the G2037R mutation had a more severe reduction in expression. For the microdeletion alleles, immunostaining results also differed from wild type, but the most consistent difference was an increase in the width of the collagen VII layer (Fig. S4) that was not observed for the targeted alleles (Fig. 4C).

### The stability of mutant collagen VII is reduced

**Fig. 5.**
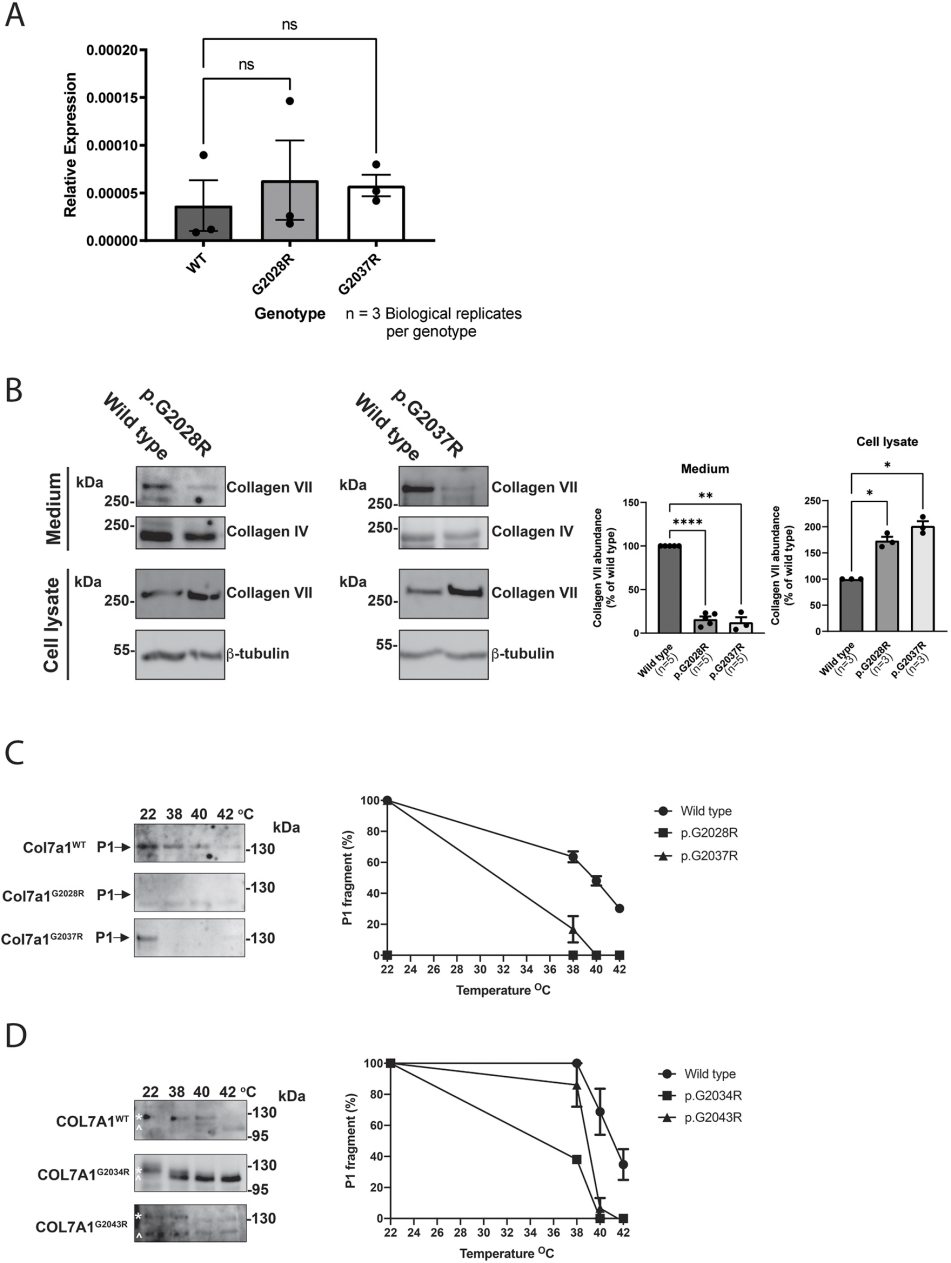
***Col7a1* changes appear to be the result of increased intracellular retention and reduced extracellular stability.** (A) qRT-PCR of *Col7a1* mRNA relative to the geometric mean of *Gapdh* and lamin A mRNA shows that there is no significant change at the mRNA level between wild-type and mutant back skin (individual data points represent biological replicates, and mean±s.e.m. are shown). Analysis by one way ANOVA; ns, not significant. (B) 3 million wild-type and mutant MEFs were cultured, and the intracellular and extracellular collagen VII protein levels were assessed from the cell lysates and culture media, respectively. Western blot (left panels) show that the amount of secreted collagen VII in the medium is reduced in both mutants, whereas the amount of intracellular collagen VII is increased in the mutants compared to wild type. These experiments were conducted three times, and quantification was performed (right panels) using the intracellular housekeeper protein, β-tubulin and the secreted protein, collagen IV, as normalisation controls for the cell lysates and culture media, respectively (individual data points represent biological replicates and mean±s.e.m. are shown). Analysis by one-way ANOVA, *P<0.05, **P<0.01, ****P<0.0001. (C) Left: limited trypsin digests of the collagen VII protein from mouse mutants show digestion at lower temperatures compared with wild-type controls. Right: quantification of digest data, showing the percentage of P1 fragment remaining at different temperatures. All digest experiments were performed three times, and each time 40 µl per lane was loaded (from a total of 200 µl precipitated medium dissolved in TBS, which was in turn derived from precipitation of 10 ml medium). Data are representative of three experiments, and mean±s.e.m. are shown. (D) Left: limited trypsin digests of the collagen VII protein from human patient samples also show decreased collagen VII stability for the p.G2034R (mouse p.G2028R) and p.G2043R (mouse p.G2037R) mutations. Asterisks indicate collagen VII collagenous P1 domain; carets indicate intermediate degradation product. Right: quantification of digest data, showing the percentage of P1 fragment remaining at different temperatures. All digest experiments were performed three times, and each time 40 µl per lane was loaded (from a total of 200 µl precipitated medium dissolved in TBS, which was in turn derived from precipitation of 10 ml medium). Data are representative of three experiments, and mean±s.e.m. are shown.

Given the changes in collagen VII staining intensity observed in mice carrying the two targeted *Col7a1* alleles, we next sought to determine via quantitative RT-PCR (qRT-PCR) whether there were any differences in *Col7a1* mRNA abundance as a result of these mutations. Using back skin as a source of RNA, we found that *Col7a1* mRNA levels were comparable across wild-type and mutant alleles (Fig. 5A; Fig. S5), suggesting that *Col7a1* transcription rates and RNA stability are not impaired by the mutations. In contrast, western blotting of mouse embryonic fibroblast (MEF) protein lysates from the two targeted mutant alleles revealed a reduction in the levels of secreted collagen VII present in the media (Fig. 5B), consistent with our immunostaining results. However, intracellular collagen VII was significantly increased in cell lysates from MEFs carrying either of the targeted alleles (Fig. 5B). Collectively, these data suggested impaired secretion of collagen VII due to misfolding and intracellular retention, but it was possible that a reduction in protein stability of the secreted mutant collagen VII might also be important.

To explore this possibility, limited trypsin digest assays were performed and showed markedly reduced stability of the collagen VII triple helix from MEFs carrying the p.G2028R and p.G2037R mutations (Fig. 5C). This was consistent with samples from patients, which also showed a decrease in collagen VII stability for the homologous p.G2034R and p.G2043R mutations (Fig. 5D).

## DISCUSSION

Here, we describe the generation of multiple novel DDEB mouse models, two of which not only carry a mutation directly homologous to the two most common mutations found in DDEB patients but also closely recapitulate the human disease. All these mutations lie within the collagen VII triple-helical region, and, together, our data reaffirm the importance of the triple helix for DDEB pathogenesis.

Among our two targeted models, mice carrying the p.G2037R allele displayed the more severe phenotype and also the greater reduction in collagen VII expression as revealed by skin immunostaining. This suggests that the level of expression of collagen VII in DDEB skin is likely to be an important factor in determining the severity of disease, and is consistent with clinical observations that RDEB patients with more severe disease are those that completely lack functional collagen VII protein. However, we cannot exclude the possibility that the glycine at position 2037 might be more structurally critical than the one at position 2028. Looking ahead, it would be interesting to determine whether the difference in severity between these two alleles is also observed between DDEB patients carrying mutations at their corresponding positions of G2034 and G2043. In practice, however, this might be difficult, because phenotypic differences in such patients are likely to be confounded by differences in genetic background and environmental exposures. Another potential avenue for further study would be to generate mice homozygous for the two targeted alleles, so as to check for a gene-dosage effect.

The models described here represent to our knowledge the first mouse models for DDEB. A rat model of DDEB (Nyström et al., 2013b) – which arose from a spontaneous glycine to aspartic acid substitution at position 1867 (in exon 69) – has previously been described by members of our team. Like our mice, the rat model displays dominantly inherited blistering and nail dystrophy. Although both the rat and mouse models will be useful for studying DDEB, we believe that our two mouse models carry several advantages over the rat model in this regard. First, our mice directly model the two most frequent human mutations seen in DDEB (van den Akker et al., 2011; Wertheim-Tysarowska et al., 2012) and, as such, carry strong construct validity (McGraw et al., 2017). Second, the common genetic aetiology between these mice and DDEB patients may make them a more useful pre-clinical model for therapeutic development. For example, a clinical trial is currently underway to test the effectiveness of an oligonucleotide that promotes skipping of exon 73 in RDEB patients (https://clinicaltrials.gov/ct2/show/NCT03605069), and such an oligonucleotide might also be worth testing in our mice. After all, the mutations in our mice are all in exon 73, which is highly conserved between humans and mice, and recent *in vitro* models suggest that oligonucleotides may also be useful for DDEB (Bornert et al., 2020). Finally, the abundance of genetic tools and experimental methods that are available for mouse-based research – as well as the ability to easily generate sufficient numbers of the animals for well-powered experimental studies – make our DDEB mice well suited to address mechanistic questions related to DDEB pathogenesis. This includes the opportunity to explore the role of inflammation in DDEB, for which we have preliminary evidence in our mice Fig. S6).

Although mouse models for other forms of EB do exist (e.g. junctional EB, EB simplex, RDEB, etc.), such animals typically demonstrate severe disease and early lethality (Bruckner-Tuderman et al., 2010; Uitto et al., 2016), which can present practical obstacles to using them as models to better understand and treat EB. In contrast, our DDEB mice showed no impairment in growth and no observable differences in survival, which – together with their relatively mild phenotype – make them well suited as an experimental model to better study EB.

Looking ahead, we therefore plan to use these mice to further explore the pathogenesis of DDEB and to investigate novel treatments for this disease. Included among our planned studies are investigations of itch and pain, which are common and significant clinical problems in patients with DDEB, although studying these symptoms in mice can be challenging.

## MATERIALS AND METHODS

### Mice and genotyping

The *Col7a1* mice used in this study were generated on a C57BL/6J background by the MAGEC laboratory at the Walter and Eliza Hall Institute (WEHI) using CRISPR-Cas9-based methods that have been previously described (Kueh et al., 2017). Specifically, to generate mice carrying the p.G2028R allele (*Col7a1*^em1Kepa^), 20 ng/µl Cas9 mRNA, 10 ng/µl sgRNA (CTGGCCCACCCGGCCTTGCG) and 40 ng/µl oligonucleotide donor (CAGGGGCCTCCTGGCCTGGCCCTGGGTGAGAGGGGCCCACCTGGCCCACCCGGCCTTGCGCGTGAACCCGGAAAGCCTGGCATTCCTGGACTCCCAGGCCGGGCTGGTGGTTCAGGGGAAGCA) were injected into the cytoplasm of fertilised one-cell-stage embryos. The same method was used to generate mice carrying the p.G2037R allele (*Col7a1*^em2Kepa^), using the sgRNA (CCAGCCCGGCCTGGGAGTCC) and oligonucleotide donor (TGAGAGGGGCCCACCTGGCCCACCCGGCCTTGCGGGGGAACCCGGAAAGCCTGGCATTCCTCGTCTTCCAGGCCGGGCTGGTGGTTCAGGGGAAGCAGGAAGGCCAGGAGAGAGGgtgagcctgggg). Twenty-four hours after injection, two-cell-stage embryos were transferred into the oviducts of pseudo-pregnant female mice. Because CRISPR-Cas9-mediated genome editing typically results in not only alleles carrying the intended targeted mutation but also alleles carrying other incidental changes, viable offspring were genotyped by next-generation sequencing to determine the range of resultant alleles. MiSeq next-generation sequencing was performed with a standard protocol with the forward primer (5′-GTGACCTATGAACTCAGGAGTCCGAGGCTGCTTTTCTTCG-3′) and reverse primer (5′-CTGAGACTTGCACATCGCAGCAACTCGTCCTCCTCCTCCTC-3′). Mice carrying the correctly targeted p.G2028R (*Col7a1*^em1Kepa^) and p.G2037R (*Col7a1*^em2Kepa^) alleles as well as three additional missense alleles containing small, in-frame deletions of six amino acids, p.I2035_G2040del (*Col7a1*^em3Kepa^), 11 amino acids, p.A2027_G2037del (*Col7a1*^em4Kepa^), and 15 amino acids, p.G2028_A2042del (*Col7a1*^em5Kepa^), were identified and continuously backcrossed with wild-type C57BL/6 mice to remove any off-target mutations (for the experiments performed in this study between two and six backcrosses had already been performed), and maintained in the animal facilities at the WEHI according to national and institutional guidelines for animal care. Subsequent genotyping of each line of mice was performed by Transnetyx, using real-time, allele-specific PCR. Both male and female animals were used in experiments.

### Characterisation of macroscopic skin changes

To identify macroscopic skin changes, mice carrying each of the novel *Col7a1* alleles and their wild-type littermates were inspected throughout the first week of postnatal life and then weekly thereafter. To document these changes, photographs were routinely taken at P1, P7, and 3, 6, 9 and 12 weeks of age using a Canon EOS 550D with a Canon Macro EFS 35 mm lens.

### Transmission EM

For light microscopic assessment of skin, mice were sacrificed at 12 weeks of age via CO_2_ inhalation. Back fur was removed with clippers followed by treatment with Nair™ (Church & Dwight, Sydney, Australia) hair removal cream as per the manufacturer's instructions to establish hairless skin, and then the underlying skin was removed and fixed in PBS/4% paraformaldehyde overnight. Skin samples were subsequently embedded in paraffin blocks, sectioned, mounted onto glass slides, stained with H&E, and finally imaged using a 40×/0.75 NA objective lens attached to an Axioplan 2 microscope (Carl Zeiss MicroImaging).

For transmission EM, mice were sacrificed at 12 weeks of age via CO_2_ inhalation. Back fur was removed with clippers followed by treatment with Nair™ hair removal cream as per the manufacturer's instructions to establish hairless skin, and then the underlying skin was removed and fixed in 2% glutaraldehyde for >2 days at 4°C, washed twice in 0.1 M cacodylate buffer and incubated for 1 h in 1% osmium tetroxide solution. After dehydration in ethanol (25, 50, 75, 90 and 100%) and propylene oxide, samples were embedded in an epoxide resin. Sections of 70 nm thickness were mounted on microscopy grids, stained with 5% uranyl acetate and Reynold's solution, and examined with an electron microscope JEM1400 (JEOL) equipped with a 2K TVIPS CCD camera TemCam216.

### Immunofluorescence

Back skin was removed from mice at 12 weeks of age as described above and embedded in optimal cutting temperature (OCT) compound prior to freezing on dry ice. Frozen sections were cut on a cryostat, transferred to pre-cooled glass slides, warmed to room temperature for 30 min, washed twice in PBS containing 0.1% Tween 20 (v/v) (PBS-T) twice for 2 min, fixed in ice-cold acetone for 5 min, air dried for 30 min, washed twice again in PBS-T, before blocking in 3% BSA/PBS-T for 20 min. Slides were then incubated overnight at 4°C in 3% BSA/PBS-T containing 1:10,000 rabbit antibody LH7.2, which recognises collagen VII (Bornert et al., 2019). On the following day, slides were washed three times in PBS-T for 5 min each, incubated in 3% BSA/PBS-T containing 1:1000 Alexa Fluor 488-conjugated goat anti-rabbit secondary antibody (Invitrogen) for 1 h at room temperature, washed again in PBS-T three times, and then cover-slipped with ProLong Gold Antifade Solution with DAPI (Invitrogen, Carlsbad, CA, USA).

Images were acquired on a Leica SP8 using a 20× HC PL APO CS2 0.75 NA objective. Excitation was provided by a pulsed 1030 nm, 750 nm or constant-wave 488 nm laser for second harmonic, DAPI and Alexa Fluor 488, respectively. Emission was collected through 400-445 nm detection gates for second harmonic and DAPI and 495-550 nm for Alexa Fluor 488. Five images per biological replicate were analysed, with three biological replicates per genotype.

### Image analysis

Quantification of anchoring fibrils in randomly selected areas and of western blots was performed using ImageJ software (National Institutes of Health). Image analysis of collagen VII staining intensity was performed in the Fiji distribution of ImageJ (Schindelin et al., 2012) with a custom-written macro (available upon request). The macro, in brief, used a user-defined region of interest of the membrane that was then used to measure the width and intensity of the staining every 1.5 µm along the length traced.

### Cell lines

Primary MEFs carrying wild-type and novel *Col7a1* alleles were created as previously described (Durkin et al., 2013), frozen in liquid nitrogen after the first passage, then subsequently expanded as needed for qRT-PCR, western blotting and trypsin digest assays.

### qRT-PCR

Back skin (30 mg) was removed and snap frozen in liquid N_2_, then crushed with a hammer on a metal plate, while cooled on dry ice between overhead projector transparencies. Skin RNA was then isolated using Trizol reagent (Invitrogen, Carlsbad, CA, USA) according to the manufacturer's instructions, and cDNA was synthesised with Superscript II reverse transcriptase (Invitrogen) and oligo(dT)15 primers (Promega, Madison, WI, USA). *Col7a1* qPCR for an amplicon spanning exons 1 and 2 was then performed using SYBRGreen Master Mix (Bioneer, Daejeon, South Korea) on a LightCycler^®^480 II (Roche, Basel, Switzerland) (Fwd primer, 5′-CTGCAGAGATCCTGATGGGA-3′; Rev primer, 5′-GATCATCACTGTACTGCACT-3′). The geometric mean of lamin A and *Gapdh* expression was used for normalisation purposes (lamin A Fwd primer, 5′-TTCGAGTGACTGTGACACTGG-3′; lamin A Rev primer, 5′-AACCCGCTGAGTACAACCT-3′; *Gapdh* Fwd primer, 5′-CAACTTTGTCAAGCTCATTTCCTG-3′; *Gapdh* Rev primer, 5′-CCTCTCTTGCTCAGTGTCCTT-3′).

### Western blotting and limited trypsin digestion assay

MEF cell lysates were extracted with NP-40 lysis buffer. Lysates were boiled in sample buffer containing 8 M urea, separated on 7% SDS-polyacrylamide gels and electrotransferred onto nitrocellulose membranes. The membranes were blocked in 5% milk in TBS-T, incubated with primary antibodies [either rabbit polyclonal murine anti-collagen VII (NC1 reactive) (Bornert et al., 2019), anti-collagen VII P1 domain (NC2-10) (Bornert et al., 2016), rabbit polyclonal anti-collagen IV (Thermo Fisher Scientific; PA5-104508) or β-tubulin (Abcam; ab6046); all diluted 1:2000] in the same blocking buffer, washed and probed with horseradish peroxidase (HRP)-conjugated secondary antibodies. The blots were developed with ECL (Fermentas Thermo Fisher Scientific) and detected using a chemiluminescence detection system (Peqlab, Erlangen, Amersham Imager 600, GE Healthcare, Freiburg, Germany).

For the limited trypsin digestion assay to assess collagen VII stability, MEFs were grown to confluence in Dulbecco's modified Eagle medium (DMEM):F12+10% foetal calf serum, after which cells were washed in PBS and serum-free DMEM:F12 with 50 µg/ml ascorbic acid added. The cells were incubated for 2 days with ascorbic acid added fresh every day. After 2 days, the media were collected and precipitated with 20% saturated ammonium sulphate for 1 h. After centrifugation, pellets were dissolved in TBS, the cell layer and extracellular matrix were extracted in NP-40 buffer, and proteins were heated for 1 min to the indicated temperatures and allowed to cool down to room temperature for 10 s. Trypsin (Serva, Heidelberg, Germany) to a total volume of 0.1% v/v was added to the protein lysates for 30 s before the reaction was stopped. The samples were then analysed by western blotting. The collagen VII P1 collagenous domain was detected using the rabbit polyclonal anti-collagen VII antibody NC2-10 (Bornert et al., 2016).

### Ethics

All mouse-related procedures were conducted in accordance with National Health and Medical Research Council guidelines and approved by the animal ethics committee at the WEHI (#2015.19 and #2019.11). Patient photos and keratinocytes isolated from biopsies were collected in the Department of Dermatology, Medical Center, University of Freiburg, after informed consent was provided as previously described (Kern et al., 2006), according to the Declaration of Helsinki and in agreement with institutional ethics regulations (166/03).

### Statistics

For comparison of anchoring fibril numbers across genotypes, one-way ANOVA with Bonferroni's multiple comparisons test was used. For comparison of *Col7a1* mRNA and protein levels between wild-type and mutant alleles, ANOVA with Dunnett's multiple comparisons test was performed. Without a pre-specified effect size, sample numbers were based on animal availability.

## Supplementary Material

Supplementary information
