# Supplementary material for: Mouse models for dominant dystrophic epidermolysis bullosa carrying common human point mutations recapitulate the human disease
Source: Dis Model Mech. 2021 Jun 4;14(6):dmm048082. doi: 10.1242/dmm.048082 (PMC8214732; doi:10.1242/dmm.048082)
Supplement: Supplementary information [file dmm-14-048082-s1.pdf]

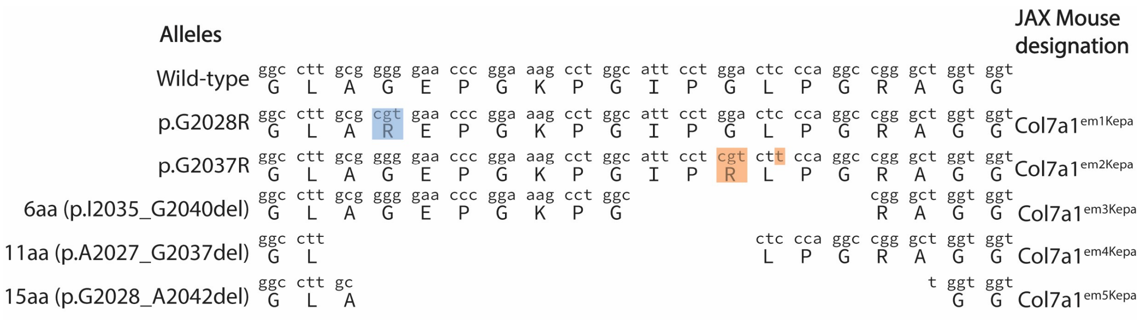

Fig. S1. Col7a1 mutations introduced via CRISPR-Cas9 editing.
In addition to the two targeted mutants, three alleles with deletions were generated, as off target effects of the CRISPR-Cas9 method. Each of these comprise small, in-frame deletions of six, eleven and fifteen amino acids (p.I2035_G2040del, p.A2027_G2037del and p.G2028_A2042del) and were designated *Col7a1*^{em3Kepa}, *Col7a1*^{em4Kepa} and *Col7a1*^{em5Kepa} respectively.

Disease Models & Mechanisms • Supplementary information

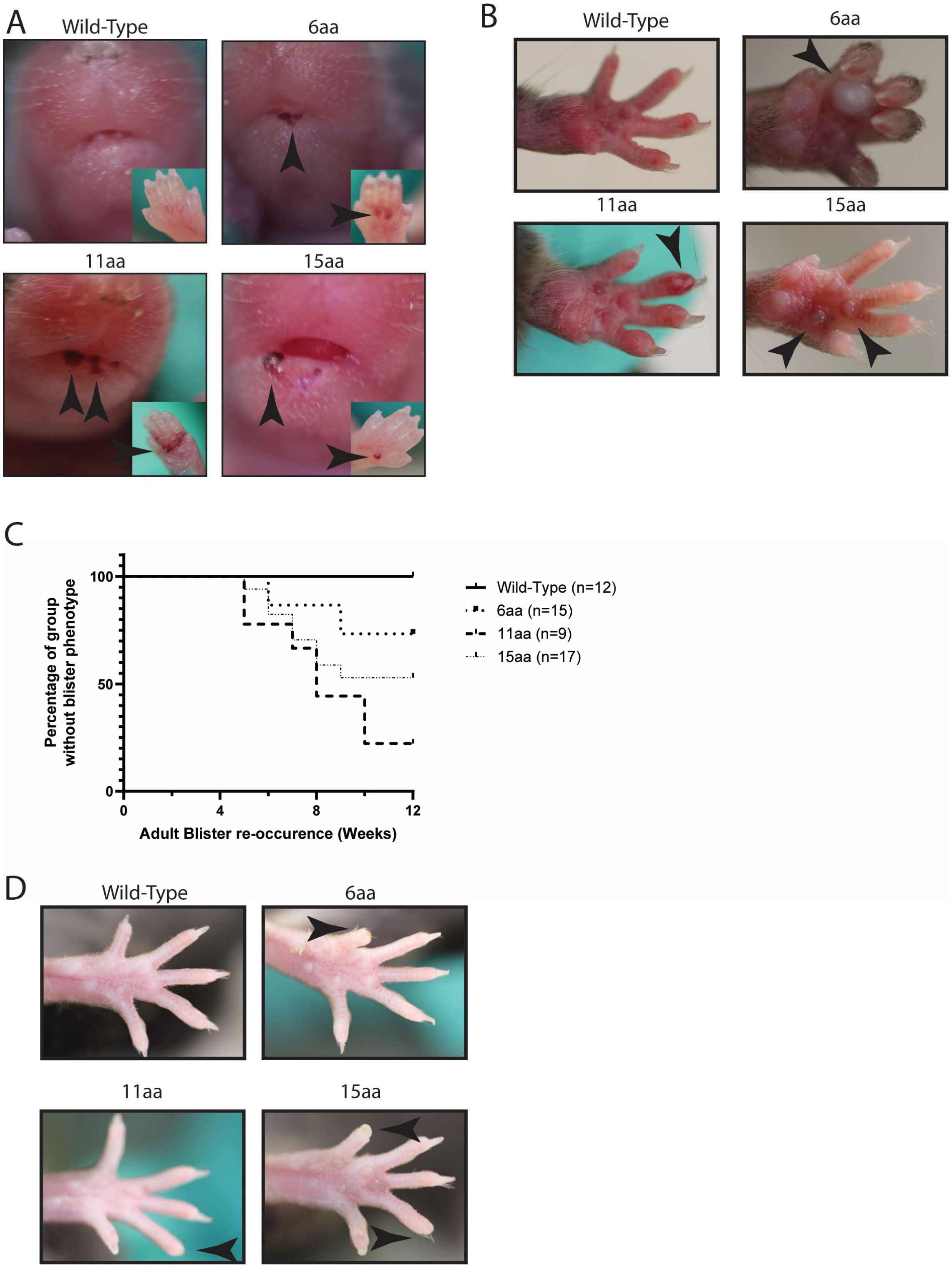

Disease Models & Mechanisms • Supplementary information

Fig. S2. In-frame Col7a1 microdeletions lead to blistering with varying penetrance of re-occurrence.

One day old mouse pups carrying the in-frame deletion alleles show varying amounts of blisters (indicated with arrows) around the mouth and on the paws (inset) (**A**). These blisters fade by the time the mice are 1 week old, and re-occur as the mice age, affecting the paws and generally becoming visible from 6-10 weeks of age (**B**). Graph showing adult blister occurrence with different patterns of onset between the in-frame deletion alleles, with the earliest occurrence at 5 weeks of age. Mice with the 6aa deletion (p.I2035_G2040del) generally show lower penetrance, while those with the 11aa deletion (p.A2027_G2037del) show greater penetrance, with most mice affected by 12 weeks of age. Those with the 15aa deletion (p.G2028_A2042del) have an intermediate phenotype (**C**). At 12 weeks of age the mice start to lose nails (indicated by arrows) (**D**).

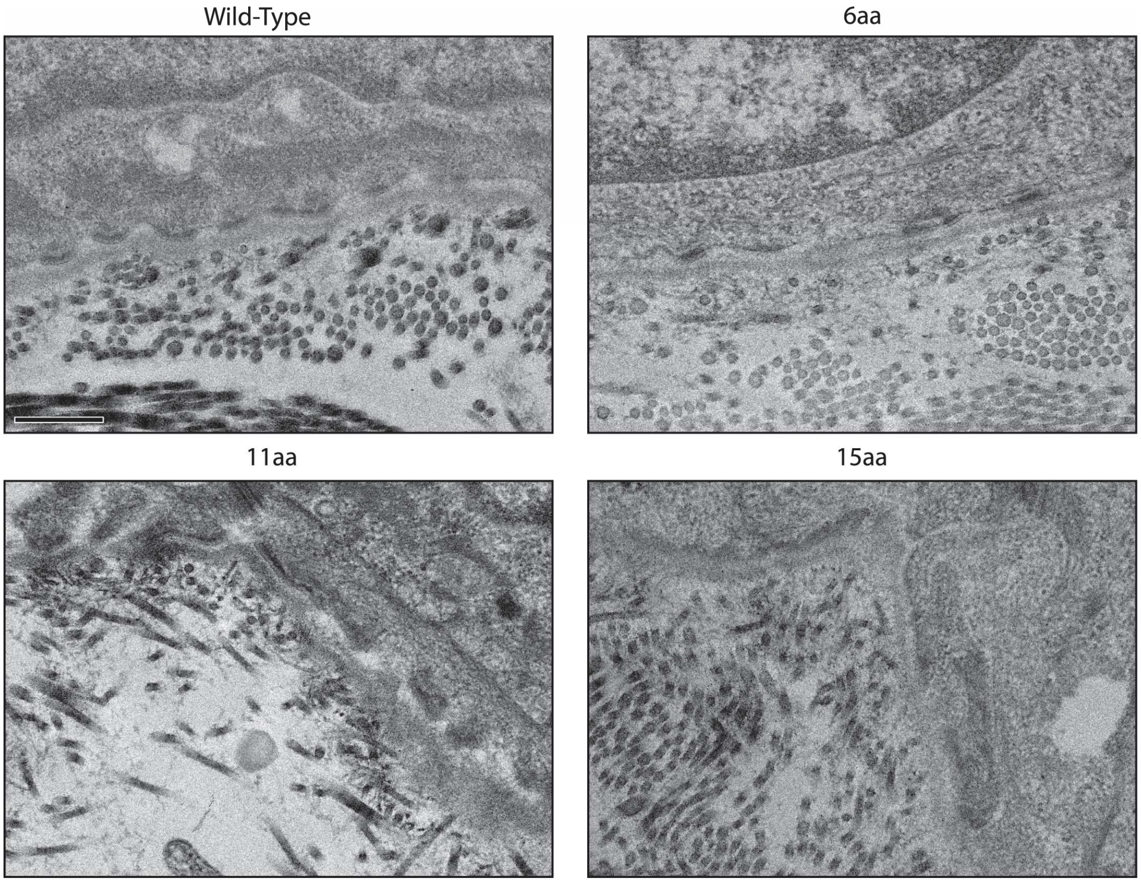

Fig. S3. Electron microscopy of skin from mice carrying in-frame Col7a1 microdeletions.
Electron microscopy of 12-week-old back skin shows possible minor disruption of anchoring fibrils within the basement layer (scale bar = 400nm)

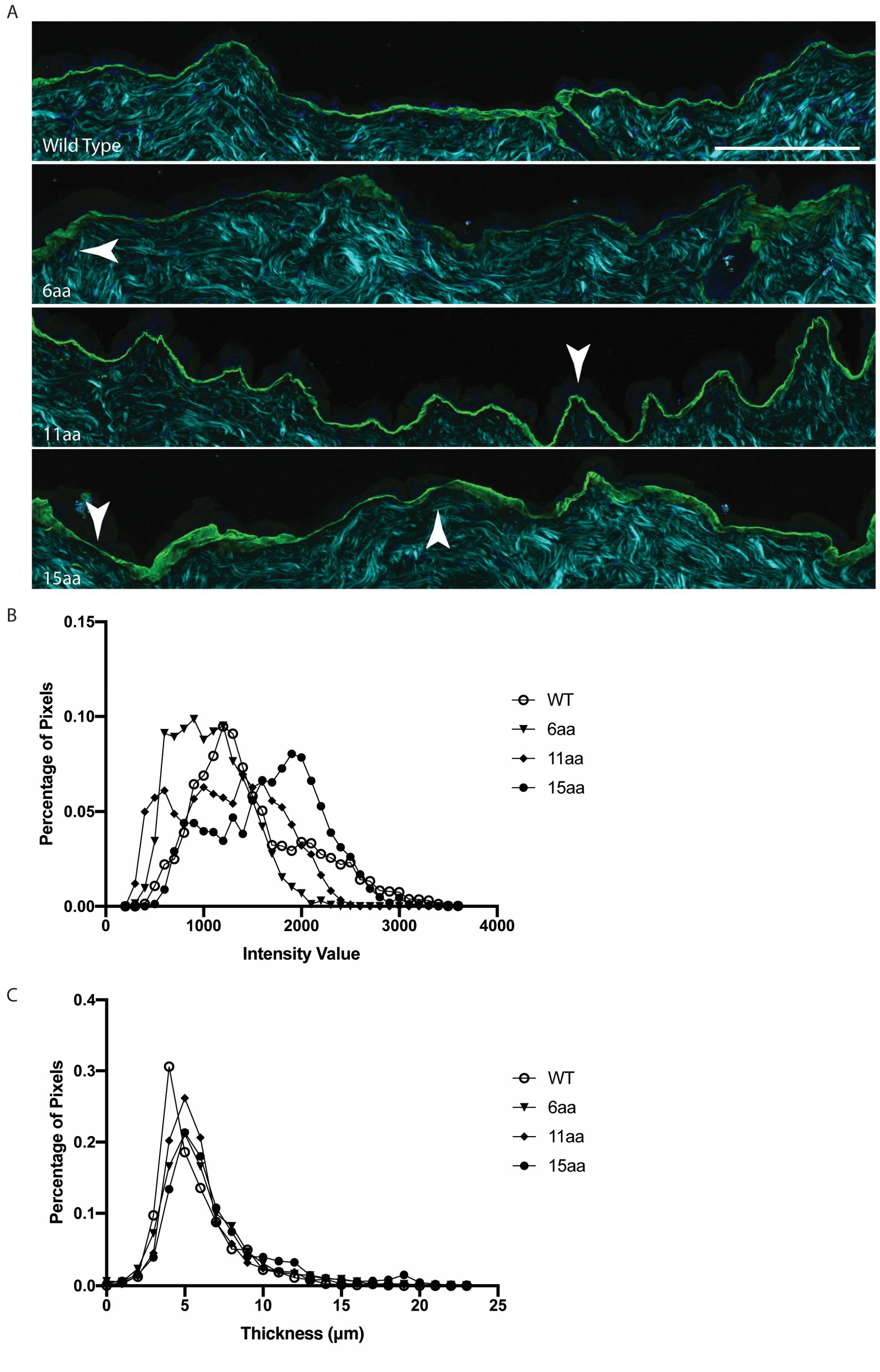

Disease Models & Mechanisms • Supplementary information

Fig. S4. Immunofluorescence staining of collagen VII in mice carrying in-frame Col7a1 microdeletions.
Immunofluorescent staining of collagen VII (green) and DAPI (dark blue) in conjunction with second harmonic imaging (cyan), which shows the structure of ordered collagens, in sections from 12-week-old mouse back skin. Changes in the intensity and thickness of collagen VII in the mutant sections are apparent when compared with wild-type skin, as is the presence of micro blisters (white arrows) below the basement membrane (scale bar: 100 µm) (**A**). Analysis of the stained sections (5 images per replicate, from 3 biological replicates per genotype) shows that the intensity of collagen VII staining varies across the mutants. Mice with the 6aa deletion showing slightly less intensity than wild-type, while those with the 11aa and 15aa deletions have an intensity that is close to or above wild-type levels (**B**). Analysis of collagen VII thickness shows that mice with the 6aa, 11aa, and 15aa deletions have slightly thicker layers of collagen VII. (**C**).

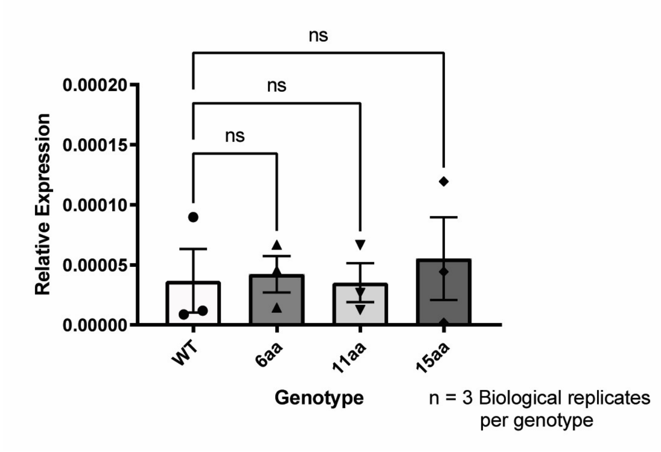

Fig. S5. qRT-PCR of *Col7a1* mRNA from skin of mice carrying in-frame *Col7a1* microdeletions.re S5.

qRT-PCR of *Col7a1* mRNA relative to the geometric mean of Gapdh and LaminA mRNA shows that there is no significant change at the mRNA level in the back skin of wild-type mice and those carrying microdeletions (individual data points represent biological replicates, and mean ± SEM are shown).

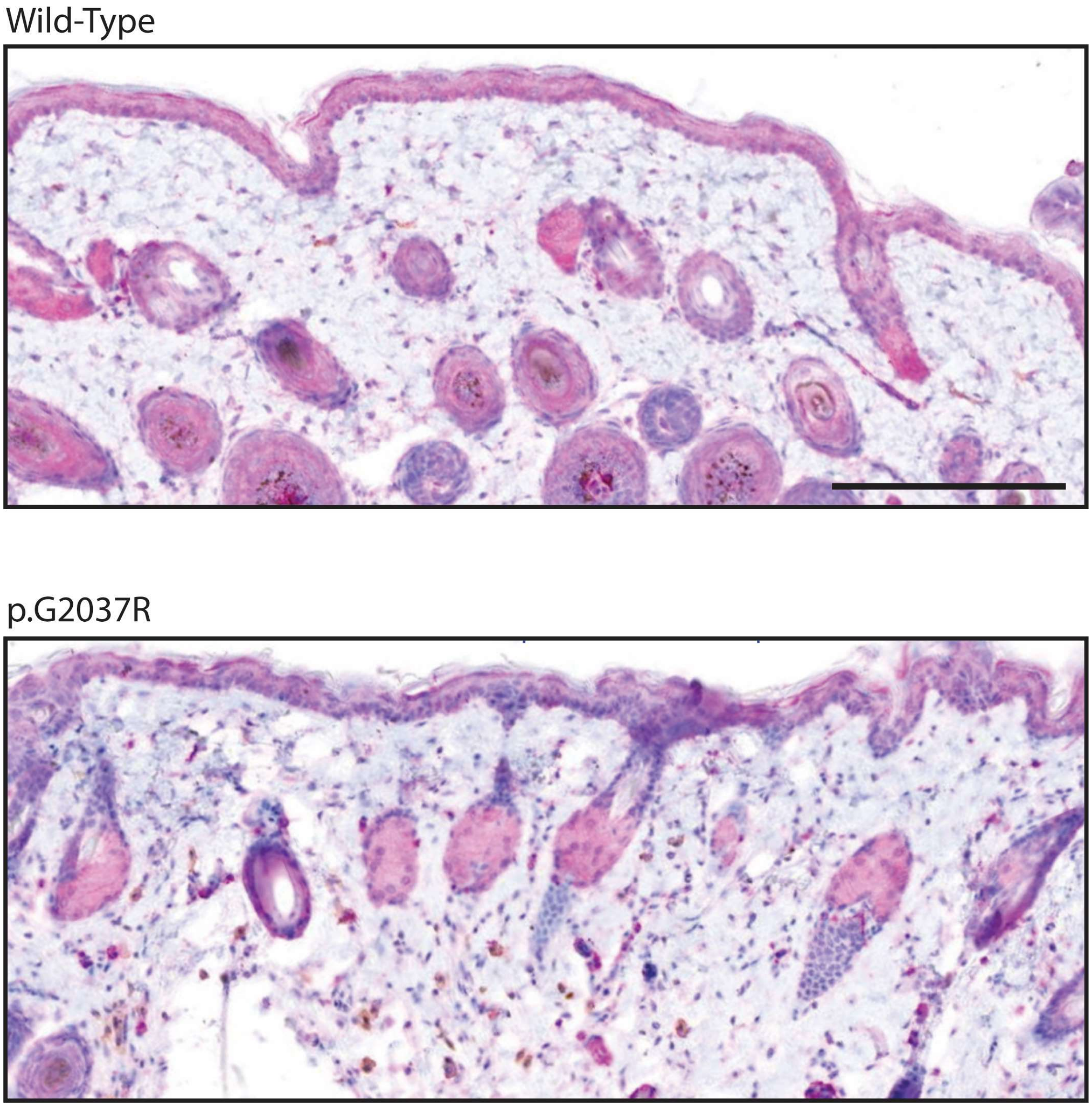

Fig. S6. Immunohistochemical staining of skin from mice carrying in-frame Col7a1 microdeletions shows an apparent increase in CD45+ and S100+ cells.
Immunohistochemical staining of formalin-fixed, paraffin-embedded hind footpad skin from wild-type and pG2037R mice showing expression of CD45 (pan-immune cell marker; shown in brown) and S100 (inflammatory marker; shown in red) with H&E counterstaining. The dermis of pG2037R animals shows an apparent increase in CD45+ and S100+ cells compared to wild-type. Scale bar = 200nm.

Table S1. Recovery of mice carrying Col7a1 mutations at weaning

Mutant allele*	Recovered wild-type mice at weaning	Recovered mutant mice at weaning	Chi-square p-value
p.G2028R	118	125	0.7
p.G2037R	217	220	0.9
6 aa deletion (p.I2035_G2040del)	140	116	0.3
11 aa deletion (p.A2027_G2037del)	163	140	0.3
15 aa deletion (p.G2028_A2042del)	113	103	0.6

* To assess for possible in utero or neonatal lethality, we determined the numbers and genotypes of mice that survived to weaning for each of our mutant strains. Consistent with our strategy of breeding a hemizygous animal with a wild type one, mice carrying the mutant alleles were all recovered at the expected ratio of around 1:1

Disease Models & Mechanisms • Supplementary information
